# Arsenic trioxide potentiates Gilteritinib-induced apoptosis in FLT3-ITD positive leukemic cells via IRE1a-JNK-mediated endoplasmic reticulum stress

**DOI:** 10.1186/s12935-020-01341-5

**Published:** 2020-06-17

**Authors:** Xiaoli Hu, Jiayi Cai, Jianyi Zhu, Wenjing Lang, Jihua Zhong, Hua Zhong, Fangyuan Chen

**Affiliations:** Department of Hematology, Ren Ji Hospital, School of Medicine, Shanghai Jiao Tong University, 160 Pujian Road, Shanghai, 200127 China

**Keywords:** Gilteritinib, ATO, Endoplasmic reticulum stress, IRE1a-JNK, FLT3-ITD

## Abstract

**Background:**

Acute myeloid leukemia (AML) patients with FMS-like tyrosine kinase 3-internal tandem duplication (FLT3-ITD) have a high relapse rate and poor prognosis. This study aims to explore the underlying mechanism of combining Gilteritinib with ATO at low concentration in the treatment of FLT3-ITD positive leukemias.

**Methods:**

We used both in vitro and in vivo studies to investigate the effects of combination of Gilteritinib with ATO at low concentration on FLT3-ITD positive leukemias, together with the underlying molecular mechanisms of these processes.

**Results:**

Combination of Gilteritinib with ATO showed synergistic effects on inhibiting proliferation, increasing apoptosis and attenuating invasive ability in FLT3-ITD-mutated cells and reducing tumor growth in nude mice. Results of western blot indicated that Gilteritinib increased a 160KD form of FLT3 protein on the surface of cell membrane. Detection of endoplasmic reticulum stress marker protein revealed that IRE1a and its downstream signal phosphorylated JNK were suppressed in Gilteritinib-treated FLT3-ITD positive cells. The downregulation of IRE1a induced by Gilteritinib was reversed with addition of ATO. Knockdown of IRE1a diminished the combinatorial effects of Gilteritinib plus ATO treatment and combination of tunicamycin (an endoplasmic reticulum pathway activator) with Gilteritinib achieved the similar effect as treatment with Gilteritinib plus ATO.

**Conclusions:**

Thus, ATO at low concentration potentiates Gilteritinib-induced apoptosis in FLT3-ITD positive leukemic cells via IRE1a-JNK signal pathway, targeting IRE1a to cooperate with Gilteritinib may serve as a new theoretical basis on FLT3-ITD mutant AML treatment.

## Background

Acute myeloid leukemia (AML) is a kind of clonal disease of hematopoietic stem cells, characterized by inhibition of differentiation and subsequent cell accumulation at various stages of incomplete maturation [1]. Although diagnosis and treatment in recent years have been improved, the overall 5-year survival rate of AML patients is still less than 30% [2]. Therapeutic response varies greatly among different individuals due to clinical heterogeneity of AML patients [3]. FMS-like tyrosine kinase 3 (FLT3) which acts as a cytokine receptor for the FLT3 ligand belongs to class III family receptor of tyrosine kinase [4]. Mutated FLT3 is one of the most frequent mutant gene in AML that occurs in approximately 30% of patients to confer a very poor prognosis [5]. There are two major groups of mutations: FLT3-internal tandem duplications (FLT3-ITDs) in the juxtamembrane (JM) domain occur in about 20% to 25% of patients [6–8]; FLT3 point mutations in the tyrosine-kinase domain (FLT3-TKD) occur in about 7% to 10% of patients [9, 10]. Our study mainly involves in FLT3-ITD which is the most common mutation of FLT3.

Several FLT3 tyrosine kinase inhibitors (TKIs) have been developed to inhibit FLT3 signaling, and the clinical trials are underway [11]. Gilteritinib is a new generation of FLT3 TKIs developed for the treatment of AML harboring FLT3 mutations [12]. Its selectivity, potency and activity against FLT3-activating mutations have been confirmed by several AML clinical trials [13]. Gilteritinib targets FLT3 with fewer side effects [14], and the common toxicities of Gilteritinib are diarrhea, fatigue and transaminitis [15]. Gilteritinib is also well tolerated and demonstrated antileukemic activity in a relapsed/refractory AML population [16]. Most recently, Gilteritinib has been approved by the Food and Drug Administration (FDA) for the treatment of adults with mutant FLT3 who have relapsed [17]. But Gilteritinib is more likely to have limited curative ability as a single reagent even used in early disease course, combination of Gilteritinib with front-line chemotherapy regimen is under study [18].

Arsenic trioxide (ATO) is a clinically established drug for the treatment of acute promyelocytic leukemia (APL) [19]. ATO rapidly degrades promyelocytic leukemia-retinoic acid receptor-alpha (PML-RARA) fusion protein and wild-type PML protein, and induces differentiation or apoptosis to exert a dual effect on APL cells [20]. ATO could induce apoptosis by acting on mitochondrial membrane [21, 22] and also by upregulating endoplasmic reticulum stress [23]. ATO is effective not only in the treatment of APL, but also in the treatment of other hematological tumors and some solid tumors (e.g., hepatocellular carcinoma [24] and gastric cancer [25, 26]).

Recently, there have been several reports about the combination of FLT3 first-generation inhibitor sorafenib with ATO [27, 28]. These studies mainly focus on ATO at high concentration to induce degradation of FLT3 protein but the cardiac toxicity caused by high concentrations of ATO has directly limited the clinical application of ATO, to explore the mechanism of its low concentration is more clinically meaningful. Most recently, it is reported that ATO at concentration of 0.5 µM could trigger the endoplasmic reticulum stress in FLT3-ITD positive cells [29]. Furthermore, few studies on Gilteritinib that is different from the first-generation broad-spectrum inhibitor sorafenib have been reported. Our study aims to focus on the newly applied Gilteritinib and to explore the novel mechanism of combining Gilteritinib with ATO at low concentration on the treatment of FLT3-ITD positive cells. The combinatorial effects of Gilteritinib plus ATO on cell proliferation, apoptosis assay, morphologic assessment, transwell assay and cell cycle analysis were examined. We also estimated the pro-apoptotic effect of inositol-requiring enzyme-1a (IRE1a) on Gilteritinib plus ATO-treated FLT3-ITD-mutated cells. The results suggested that ATO at low concentration could activate IRE1a-JNK signal pathway to potentiate Gilteritinib-induced apoptosis. Thus, targeting IRE1a to combine with Gilteritinib may serve as a new treatment on AML with FLT3-ITD mutation.

## Materials and methods

### Cell culture and reagents

The MV4-11, MOLM13, THP1 and HL60 cell lines were obtained from the Cell Bank of the Chinese Academy of Sciences (Shanghai, China). FLT3-ITD mutant cell lines MV4-11 and MOLM13 were cultured in IMDM (Gibco, CA, USA) supplemented with 10% fetal bovine serum (FBS) (Invitrogen, Carlsbad, CA, USA) and the leukemic cell lines THP1 and HL60 were cultured in RPMI 1640 (Gibco, CA, USA) supplemented with 10% FBS at 37 °C in 5% CO_2_. ATO was purchased from Beijing SL Pharmaceutical Co., Ltd. Gilteritinib was obtained from Medchemexpress. Dimethyl sulfoxide (DMSO), tunicamycin (TM) and bovine serum albumin (BSA) were obtained from Sigma-Aldrich. Gilteritinib, ATO and TM were dissolved in DMSO to produce a stock solution and stored at − 20 °C for cell experiment. The above stock solutions were further diluted to the appropriate concentrations in cell culture medium before experiments. Anti-FLT3, anti-phosphorylated-FLT3, anti-Human Recombinant Protein (USP10), anti-signal transducers and activators of transcription 5 (STAT5), anti-phosphorylated-STAT5, anti-extracellular regulated protein kinases (ERK), anti-phosphorylated-ERK, anti-protein kinase B (AKT), anti-phosphorylated-AKT, anti-glucose regulated protein 94 (GRP94), anti-PKR-like ER kinase (PERK), anti-activating transcription factor 6 (ATF6), anti-IRE1a, anti-c-Jun NH2-terminal kinase (JNK), anti-phosphorylated-JNK, anti-bcl2-associated X protein (BAX) and anti-B-cell lymphoma-2 (BCL-2) antibodies were obtained from Cell Signaling Technology. Anti-glyceraldehyde-3-phosphate dehydrogenase (GAPDH) antibody was purchased from Abmart. Lipofectamine 2000 was purchased from Invitrogen Corporation.

### Growth inhibition assay

MV4-11, MOLM13, THP1 and HL60 cells were treated with various concentrations (0–20 nM) of Gilteritinib for 24 h. MV4-11 and MOLM13 cells were treated with various concentrations of Gilteritinib (0–20 nM) alone, ATO (0–4 μM) alone or Gilteritinib (0–20 nM) plus ATO (0–4 μM) for 48 h. The control group was treated with DMSO. After treatment, an aliquot of the treated-cell suspension (100 μL) was seeded into each well of 96-well plate. 10 μL CCK-8 stock solution was added to each well and incubated for 2 h at 37 °C. The plates were measured at an absorbance of 450 nm by a microplate reader (Multi-scan FC, Thermo Fisher, USA).

### Cell morphology

MV4-11 and MOLM13 cells were treated with DMSO, Gilteritinib (2.5 nM) alone, ATO (0.5 µM) alone or Gilteritinib (2.5 nM) plus ATO (0.5 µM) for 48 h respectively. The cells were centrifuged onto slides by cytospin and stained with Wright–Geimsa. Images were captured using a light microscope (1000×, magnification) for morphological observations.

### Transwell experiments

The invasive ability of cells was estimated using a Matrigel coated transwell chamber with an 8-μm pore size membrane (R3NA43983, Millipore, Billerica, MA, USA) as described previously [30].

MV4-11 and MOLM13 cells were treated with DMSO, Gilteritinib (2.5 nM) alone, ATO (0.5 µM) alone or Gilteritinib (2.5 nM) plus ATO (0.5 µM) for 48 h respectively. Then the cells were centrifuged to discard culture medium and resuspended in serum-free culture medium. The treated-cell suspension was added into transwell’s upper chamber; the culture medium containing 10% FBS was added into the bottom chamber. After incubation for 24 h, the cells attached to the lower surface were fixed in 100% methanol and stained with 1% crystal violet.

### Apoptosis assay

Cell apoptosis was assessed by flow cytometry using an Annexin V-PI Kit (Nanjing Keygen Biotech. Co. Ltd., Nanjing, China) according to the instructions. MV4-11 and MOLM13 cells were treated with DMSO, Gilteritinib (2.5 nM) alone, ATO (0.5 µM) alone or Gilteritinib (2.5 nM) plus ATO (0.5 µM) for 48 h and treated with DMSO, Gilteritinib (2.5 nM) alone, TM (0.2 µM) alone or Gilteritinib (2.5 nM) plus TM (0.2 µM) for 24 h. The cells were washed in phosphate buffer saline (PBS) and resuspended in binding buffer, and then stained with 5 µL propidium iodide (PI) and 5 µL Annexin V-Fluorescein Isothiocyanate (FITC) at room temperature for 15 min. The samples were examined by flow cytometry using a FACS Calibur system (BD Biosciences, Franklin Lakes, NJ, USA) followed by apoptotic cells analysis with Cell-Quest Pro software (BD Biosciences, Franklin Lakes, NJ, USA).

### Cell cycle analysis

MV4-11 cells in six-well culture plates were treated with DMSO, Gilteritinib (2.5 nM) alone, ATO (0.5 µM) alone or Gilteritinib (2.5 nM) plus ATO (0.5 µM) for 48 h respectively. The treated-cells were washed with PBS, then fixed with 70% ethanol at 4 °C for more than 24 h. Cells were incubated with propidium iodide (Nanjing Keygen, China) for 15 min at room temperature and examined by flow cytometry using a FACS Calibur system (BD Biosciences, Franklin Lakes, NJ, USA).

### Transfection of small interfering RNA (siRNA)

The MV4-11 and MOLM13 cells were seeded into a 6-well plate at a density of 5 × 10^5^ cells/well. The siRNA was synthesized from GenePharma Company (Shanghai, China). The MV4-11 and MOLM13 cells were transiently transfected with IRE1a siRNA (IRE1a siRNA#1 sense: 5′-CAGACAGACCUGCGUAAAUUCTT-3′; antisense: 5′-GAAUUUACGCAGGUCUGUCUGTT-3′. IRE1a siRNA#2 sense: 5′-AUGGAGCUGAGGGCACAAUUGTT-3′; antisense: 5′-CAAUUGUGCCCUCAGCUCCAUTT-3′); or scramble siRNA (sense: 5′-UUCUCCGAACGUGUCACGUTT-3′; antisense: 5′-ACGUGACACGUUCGGAGAATT-3′) as negative control. IRE1a siRNA or the negative control siRNA and Lipofectamine 2000 (Invitrogen) were added to OptiMEM (Invitrogen) to incubate for 20 min at room temperature. The mixture was added to MV4-11 and MOLM13 cells in 6-well plate after incubation. After siRNA transfection overnight, cells were either collected for knockdown validation or treated with DMSO or Gilteritinib (2.5 nM) plus ATO (0.5 µM) at 37 °C for an additional 48 h for flow cytometry and western blot.

### Real-time reverse transcription-polymerase chain reaction (RT-qPCR)

RNAs extraction and RT-PCR assays were performed as previously [31]. Total RNA was extracted using Trizol (Invitrogen, Carlsbad, CA, USA) and the RNA was converted into cDNA using the PrimeScript™ RT reagent Kit (Takara Bio Inc, Shiga, Japan) via the first-strand synthesis system (Thermo Scientific, USA). RT-PCR was performed following the standard protocol on ABI 7500fast with SYBR Premix Ex Taq reagent kit (Takara Bio Inc, Shiga, Japan). The sequences of the RT-PCR primers were as follows: IRE1a sense: CACAGTGACGCTTCCTGAAAC, antisense: GCCATCATTAGGATCTGGGAGA; GAPDH sense:GGAGCGAGATCCCTCCAAAAT, antisense: GGCTGTTGTCATACTTCTCATGG.

### Western blot analysis

After treatment, MV4-11 and MOLM13 cells were harvested and lysed in Radio-Immunoprecipitation Assay (RIPA) lysis buffer (Cell Signaling Technology, Beverly, MA, USA) on ice for 30 min according to the manufacturer’s protocol. Then the cells were centrifuged at 12000*g* for 15 min at 4 °C and the supernatant was collected. Bicinchoninic acid (BCA) reagent (Thermo Scientific, Waltham, MA, USA) was used to determine the protein concentration. Equal amounts (20 µg) of protein extract were applied to 10% SDS-polyacrylamide gels and transferred to a polyvinylidene fluoride (PVDF) membrane (Bio Rad, Hercules, CA, USA). Then, the membranes were incubated with primary antibodies overnight at 4 °C. After three washes with Tris Buffered Saline Tween (TBST) buffer, membranes were incubated with secondary antibodies (CST, Beverly, MA, USA) for 2 h. The target protein bands were examined by an ECL kit (Millipore, Billerica, MA, USA).

### Tumor xenograft in nude mice

Six-week-old female nude mice were purchased from the SLAC (Shanghai, China). All the animal experiments were agreed by the Animal Care and Ethical Committee of Ren Ji Hospital Affiliated to Shanghai Jiaotong University. Xenograft tumors were generated by injecting subcutaneously 1 × 10^7^ MV4-11 cells in 100 µL of PBS on left flank in nude mice. When the tumors reached 100 mm^3^ in size, animals which divided randomly into four group (5 mice of each group) were treated daily with Gilteritinib (10 mg/kg/day, orally) and/or ATO (1 mg/kg/day, intraperitoneally) or vehicle for 2 weeks. Tumors were measured with a caliper and volume was calculated by the formula: V = A × B^2^/2 (A is the larger diameter and B is the smaller diameter). After treatment for 2 weeks, the tumors were removed from the nude mice for further experiments.

### TUNEL staining

The distribution of apoptotic cells in tumor was measured by TUNEL assay kit (In Situ Cell Death Detection kit; Roche Diagnostics GmbH, Mannheim, Germany) according to the manufacturer’s protocol. The deparaffinized sections were treated with xylene and rehydrated in graded alcohol. After two washes with PBS, the sections were incubated with the mixture of prepared TUNEL reagent at 37 °C in the humidified chamber away from light for 60 min. Green-fluorescence in the nuclei was visualized as apoptosis. TUNEL-positive cells were imaged under a fluorescence microscope (Nikon, Tokyo, Japan).

### Statistical analysis

All data were expressed as the mean ± standard deviation. For all analyses, comparisons between various conditions were performed using an unpaired t-test. P < 0.05 was considered statistically significant. All statistical analyses were performed using the SPSS 20.0 software program (Statistical Package for Social Science, SPSS Inc. Chicago, IL., USA). Curves and histograms were constructed using GraphPad Prism 5.0 software (GraphPad Software, Inc., La Jolla, CA, USA).

## Results

### FLT3-ITD-mutated cell lines are more sensitive to Gilteritinib

**Fig. 1:**
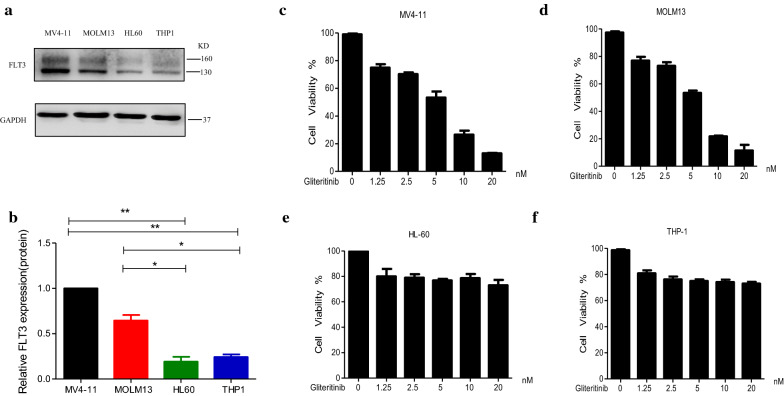
FLT3-ITD-mutated cell lines are more sensitive to Gilteritinib. **a**, **b** Western blot analysis of FLT3 in MV4-11, MOLM13, HL60 and THP1 cells. Data are shown as mean ± SD.*P < 0.05, **P < 0.01. **c**–**f** Cell viability was measured by CCK8 kit. MV4-11, MOLM13, HL60 and THP1 cells were treated with Gilteritinib (0–20 nM) for 24 h. DMSO treated cells served as a control. Data are shown as mean ± SD

In an initial screen, we first examined the expression of total FLT3 protein in FLT3-WT cells (THP1 and HL60) and FLT3-ITD mutant cells (MV4-11 and MOLM13) by western blot and compared the therapeutic effects of Gilteritinib between FLT3-WT cells (THP1 and HL60) and FLT3-ITD mutant cells (MV4-11 and MOLM13). Among these leukemic cell lines, higher expression of FLT3 was detected in FLT3-ITD-mutated MV4-11 and MOLM13 cell lines (Fig. 1a, b). The leukemic cell lines including MV4-11, MOLM13, THP1 and HL60 were treated with increasing concentrations of Gilteritinib (0–20 nM) and the anti-proliferative effect of Gilteritinib was examined by CCK-8 kit. The control group was treated with DMSO. Proliferation assay demonstrated that FLT3-ITD mutant cells (MV4-11 and MOLM13) were more sensitive to Gilteritinib compared with FLT3-WT cells (THP1 and HL60) (Fig. 1c–f). This corroborated that Gilteritinib exhibited a greater effect on FLT3-ITD mutant leukemic cells.

### Synergistic effects of Gilteritinib and ATO in FLT3-ITD-mutated cell lines

**Fig. 2:**
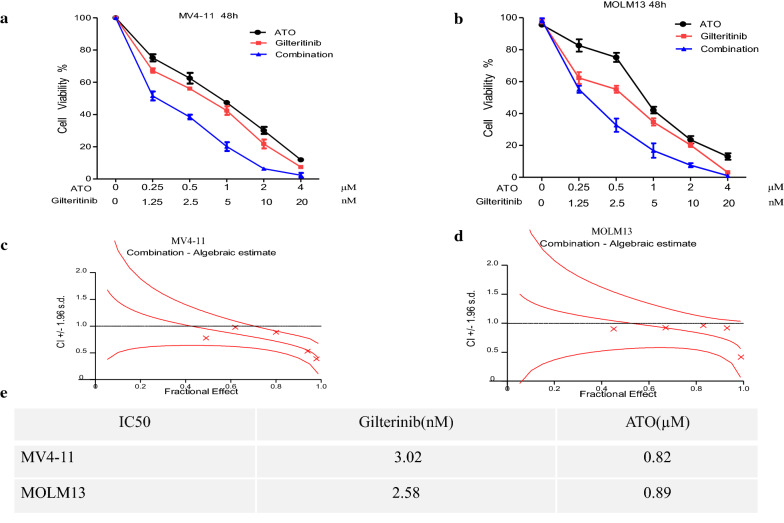
Gilteritinib synergizes with ATO to reduce proliferation of FLT3-ITD cell lines. **a**, **b** MV4-11 and MOLM13 cells treated with Gilteritinib (0–20 nM), ATO (0–4 μM) either alone or in combination for 48 h were detected by CCK8 assay. DMSO treated cells served as a control. **c**, **d** Curve of combination index (CI) analyzed by Calcusyn Software was shown. **e** IC50 was listed according to CCK8 detection

To evaluate combinatorial effects on cell proliferation, FLT3-ITD positive cells were treated with increasing concentrations of Gilteritinib (0–20 nM) alone, ATO (0–4 μM) alone, or the combination for 48 h. The control group was treated with DMSO. Treatment with Gilteritinib plus ATO achieved a further inhibition of proliferation in MV4-11 and MOLM13 cell lines compared with Gilteritinib alone or ATO alone (Fig. 2a, b). The combinatorial effect on cell proliferation was assessed by the Chou-Talalay method to calculate combinatorial index (CI). Synergistic effects were observed for the combination of Gilteritinib with ATO against FLT3-ITD-mutated cells with CI values < 1 (Fig. 2c, d) indicating synergy. The 50% inhibitory concentration values (IC50) of Gilteritinib in MV4-11 and MOLM13 cell lines for 48 h was 3.02 nM and 2.58 nM, respectively; IC50 of ATO in MV4-11 and MOLM13 cell lines for 48 h was 0.82 µM and 0.89 µM (Fig. 2e).

**Fig. 3:**
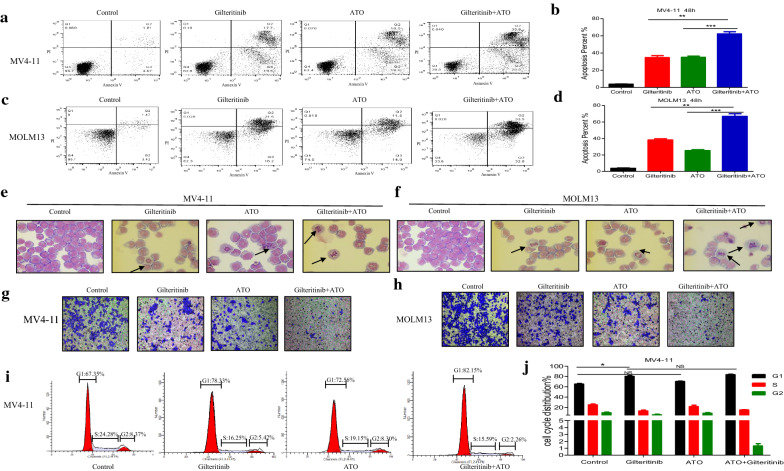
Synergistic effects of Gilteritinib and ATO in FLT3-ITD cell lines. **a**–**d** MV4-11 and MOLM13 cells were treated with Gilteritinib (2.5 nM) and/or ATO (0.5 µM) for 48 h and cell apoptosis was determined by flow cytometry. DMSO treated cells served as a control. Data are shown as the mean ± SD. **P < 0.01, ***P < 0.001. **e**, **f** Cellular morphology of the MV4-11 and MOLM13 cells treated with Gilteritinib (2.5 nM) and/or ATO (0.5 µM) for 48 h (Wright–Geimsa staining; magnification, ×1000). Typical features of apoptotic cells were marked by arrows. **g**, **h** Transwell assay of MV4-11 and MOLM13 cells treated with Gilteritinib (2.5 nM) and/or ATO (0.5 µM) for 48 h was performed. **i**, **j** Cell cycle analysis of MV4-11 cells treated with Gilteritinib (2.5 nM) and/or ATO (0.5 µM) for 48 h was determined by flow cytometry. Data are shown as the mean ± SD. *P < 0.05

We found that the addition of ATO at low concentration to Gilteritinib could further inhibit the proliferation of cells. To explore the synergistic effect of combining Gilteritinib with ATO at low concentration in both FLT3-ITD-mutated cells, apoptosis assay, morphologic assessment, transwell assay and cell cycle analysis were performed. A fixed concentration of each drug (2.5 nM Gilteritinib and 0.5 µM ATO) was chosen. The combination of Gilteritinib with ATO could significantly increase the apoptotic proportion of MV4-11 and MOLM13 cells compared with Gilteritinib alone or ATO alone (Fig. 3a–d). Typical features of apoptotic cells were visualized using light microscopy and an increase of typical apoptotic cells was observed following Gilteritinib plus ATO treatment (Fig. 3e, f). Cells treated with Gilteritinib plus ATO passing through the transwell compartment were significantly decreased according to the transwell assay in both FLT3-ITD-mutated cells (Fig. 3g, h). Combination therapy could dramatically reduce ability of invasion in MV4-11 and MOLM13 cells. Gilteritinib monotherapy could significantly induce cell cycle arrest with an increased fraction of MV4-11 cells in G1 phase and Gilteritinib plus ATO treatment upregulated G1 phase and downregulated G2 phase compared with Gilteritinib monotherapy but without statistics difference (Fig. 3i, j).

### Modulation of downstream targets of FLT3 activation by Gilteritinib plus ATO treatment

**Fig. 4:**
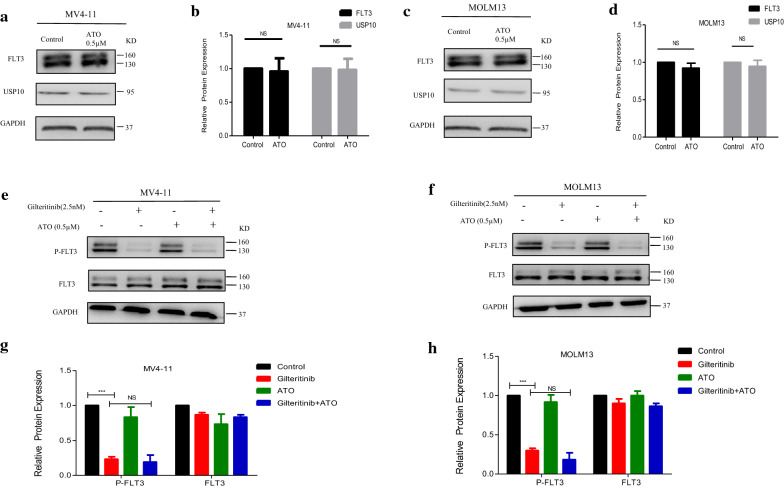
ATO at concentration of 0.5 µM has little effect on degradation of FLT3 protein. **a**–**d** MV4-11 and MOLM13 cells were treated with ATO (0.5 µM) for 48 h and protein levels of FLT3 and USP10 were determined by western blot. Data are shown as the mean ± SD. **e**–**h** MV4-11 and MOLM13 cells were treated with Gilteritinib (2.5 nM) and/or ATO (0.5 µM) for 48 h and protein levels of P-FLT3 and FLT3 were determined by western blot. Data are shown as the mean ± SD, ***P < 0.001. *P-FLT3* phosphorylated-FLT3

Previous reports had suggested that ATO could degrade FLT3 protein by downregulating USP10 to inhibit FLT3-ITD positive cells, but ATO at low concentrations (0.5 µM) failed to downregulate USP10 and could not induce degradation of total FLT3 (Fig. 4a–d) and modulation of 160KD or 130KD FLT3 (Additional file 1: Figure S1A, B). There may be a mechanism different from previous reports. Gilteritinib as a new type of inhibitor exhibited a strong inhibition on the phosphorylated FLT3 even at a low concentration of 2.5 nM (Fig. 4e–h), but had little effect on total FLT3 (Fig. 4e–h) and USP10 (Additional file 1: Figure S2A–D). Combination treatment did not further reduce the level of FLT3 activation (phosphorylated FLT3), and failed to affect the total protein level of FLT3 (Fig. 4e–h).

**Fig. 5:**
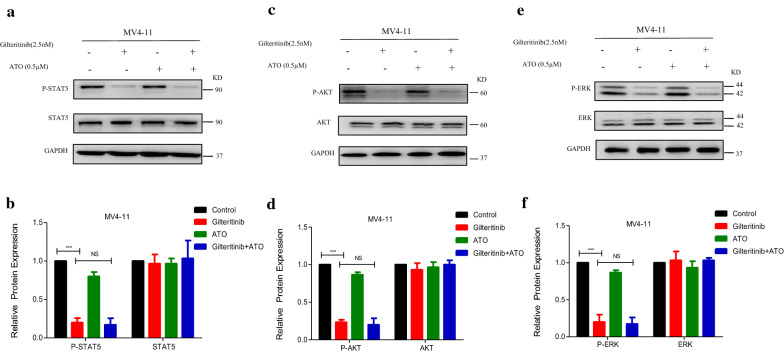
Modulation of downstream targets of FLT3 activation by Gilteritinib plus ATO treatment in MV4-11 cells. **a**–**f** MV4-11 cells were treated with Gilteritinib (2.5 nM) and/or ATO (0.5 µM) for 48 h and protein levels of P-STAT5, STAT5, P-AKT, AKT, P-ERK and ERK were determined by western blot. DMSO treated cells served as a control. Data are shown as mean ± SD for three separate experiments, ***P < 0.001. *P-STAT5* phosphorylated-STAT5, *P-AKT* phosphorylated-AKT, *P-ERK* phosphorylated-ERK

**Fig. 6:**
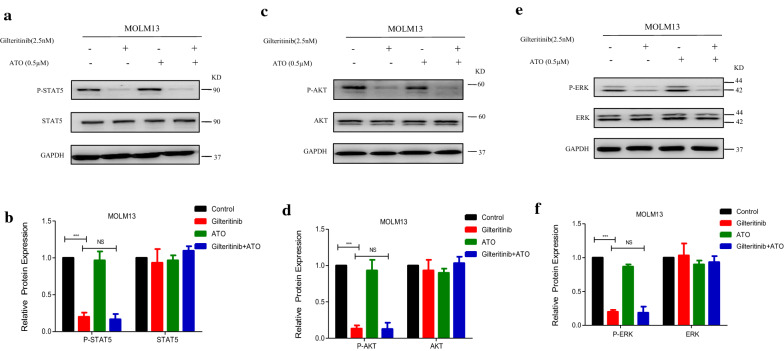
Modulation of downstream targets of FLT3 activation by Gilteritinib plus ATO treatment in MOLM13 cells. **a**–**f** MOLM13 cells were treated with Gilteritinib (2.5 nM) and/or ATO (0.5 µM) for 48 h and protein levels of P-STAT5, STAT5, P-AKT, AKT, P-ERK and ERK were determined by western blot. DMSO treated cells served as a control. Data are shown as mean ± SD for three separate experiments, ***P < 0.001. *P-STAT5* phosphorylated-STAT5, *P-AKT* phosphorylated-AKT, *P-ERK* phosphorylated-ERK

Mutant FLT3-ITD could activate STAT5, AKT and ERK pathways, all of which are downstream targets of FLT3 activation [32]. We detected whether treatment with ATO plus Gilteritinib at a fixed concentration could inhibit these abnormal pathways. MV4-11 and MOLM13 cells were treated with DMSO, Gilteritinib (2.5 nM) alone, ATO (0.5 µM) alone, or Gilteritinib (2.5 nM) plus ATO (0.5 µM) (combination) for 48 h. Gilteritinib significantly inhibited STAT5 phosphorylation (Figs. 5a, b, 6a, b), AKT phosphorylation (Figs. 5c, d, 6c, d) and ERK phosphorylation (Figs. 5e, f, 6e, f) in both cells with no effect on total expression of STAT5 (Figs. 5a, b, 6a, b), AKT (Figs. 5c, d, 6c, d) and ERK (Figs. 5e, f, 6e, f), the addition of ATO to Gilteritinib displayed no further inhibition (Figs. 5a–f, 6a–f). The combination of Gilteritinib with ATO could increase apoptosis, but the inhibition on phosphorylated abnormal pathways activated by FLT3-ITD mutation was not observed with improvement upon combination therapy. In a word, combination therapy showed no synergistic effects on inhibition of expression of FLT3 and its downstream targets, it suggested that other mechanisms may be involved in combination of the two drugs at a fixed concentration.

### Addition of ATO to Gilteritinib activates IRE1a-JNK signal pathway to induce apoptosis

**Fig. 7:**
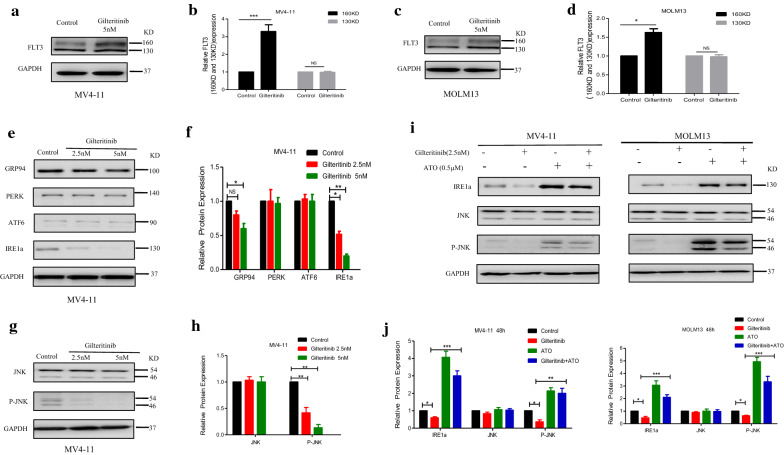
Addition of ATO to Gilteritinib activates IRE1a-JNK pathway to induce apoptosis. **a**–**d** MV4-11 and MOLM13 cells were treated with Gilteritinib (5 nM) for 48 h and protein levels of FLT3 were determined by western blot. Data are shown as the mean ± SD. *P < 0.05, ***P < 0.001. **e**, **f** MV4-11 cells were treated with Gilteritinib (2.5 nM or 5 nM) for 48 h and protein levels of GRP94, PERK, ATF6 and IRE1a were determined by western blot. Data are shown as the mean ± SD. *P < 0.05, **P < 0.01. **g**, **h** MV4-11 cells were treated with Gilteritinib (2.5 nM or 5 nM) for 48 h and protein levels of JNK and P-JNK were determined by western blot. Data are shown as the mean ± SD. **P < 0.01. **i**, **j** MV4-11 and MOLM13 cells were treated with Gilteritinib (2.5 nM) and/or ATO (0.5 µM) for 48 h and protein levels of IRE1a, JNK and P-JNK were determined by western blot. Data are shown as the mean ± SD. *P < 0.05, **P < 0.01, ***P < 0.001. *P-JNK* phosphorylated-JNK

Based on the strong apoptotic effect observed for the combination therapy in FLT3-ITD-mutated cells, we further investigated the molecular mechanism underlying the synergy. FLT3 exists in endoplasmic reticulum in the form of 130KD and on cell membrane in the form of 160KD [33]. Gilteritinib (2.5 nM) had little effect on total FLT3(160KD and 130KD), but the expression of 160KD FLT3 was observed with a slight increase under treatment with Gilteritinib (2.5 nM) (Fig. 4e–h), a significant change was found only in MOLM13 (Additional file 1: Figure S1C, D). Gilteritinib (5 nM) was used to confirm this change and Gilteritinib (5 nM) increased the expression of 160KD FLT3 on cell surface in both MV4-11 and MOLM13 cells with statistic significance at 48 h (Fig. 7a–d). An increase of mature, complex glycosylated form (160KD) of FLT3 on the surface of cell membrane which transferred from the endoplasmic reticulum in the form of 130KD was related to the decreased endoplasmic reticulum stress. We detected the endoplasmic reticulum stress-related proteins in MV4-11 cells treated with Gilteritinib (2.5 nM and 5 nM) for 48 h. GRP94, as an endoplasmic reticulum stress marker, was observed a decreased protein level to indicate the inactivation of endoplasmic reticulum stress (Fig. 7e, f). Three distinct signaling pathways are activated by PERK, ATF6, and IRE1a that are triggered in response to endoplasmic reticulum stress [34, 35]. Protein levels of PERK, ATF6, and IRE1a were tested to further explore the status of endoplasmic reticulum stress under treatment of Gilteritinib. Gilteritinib decreased IRE1a levels, while PERK and ATF6 were not changed significantly (Fig. 7e, f). Meanwhile, a decreased level of phosphorylated JNK which was the downstream targets of IRE1a was observed (Fig. 7g, h). Endoplasmic reticulum stress-activated IRE1a are proved to be involved in apoptosis in several cancers [36]. Endoplasmic reticulum stress triggered by FLT3 in the form of 130KD in endoplasmic reticulum to promote apoptosis was blocked by an increase of the expression of 160KD FLT3 on cell surface with treatment of Gilteritinib. The decreased expression of IRE1a and phosphorylated JNK indicated that inactivation of endoplasmic reticulum stress was induced by Gilteritinib.

In MV4-11 and MOLM13 cells, it was further verified that Gilteritinib was defective to activate endoplasmic reticulum stress, and ATO could significantly increase the expression of IRE1a and phosphorylated JNK to upregulate endoplasmic reticulum stress at low concentration of 0.5 µM (Fig. 7i, j). Thus, Gilteritinib decreased the expression of IRE1a and phosphorylated JNK in FLT3-ITD-mutated cells but the downregulation trend was rescued by cotreatment with ATO (Fig. 7i, j).

### Knockdown of IRE1a diminishes the synergism of Gilteritinib plus ATO combination treatment of FLT3-ITD-mutated cells

**Fig. 8:**
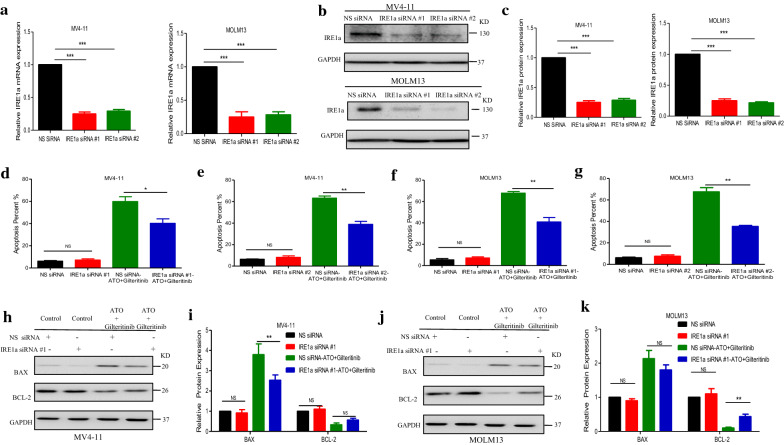
Knockdown of IRE1a diminishes the synergism of Gilteritinib plus ATO combination treatment of FLT3-ITD-mutated cells. **a** Knockdown of IRE1a in MV4-11 and MOLM13 cells was confirmed by RT-qPCR. Data are shown as mean ± SD. ***P < 0.001. **b**, **c** Knockdown of IRE1a in MV4-11 and MOLM13 cells was confirmed by western blot. Data are shown as mean ± SD. ***P < 0.001. **d**–**g** Cell apoptosis in MV4-11 and MOLM13 cells with nonsilencing (NS) siRNA or IRE1a-targeted siRNAs following treatment with Gilteritinib (2.5 nM) plus ATO (0.5 µM) for 48 h was detected using flow cytometry. Data are shown as mean ± SD. *P < 0.05, **P < 0.01. **h**–**k** Expression of BAX and BCL-2 in MV4-11 and MOLM13 cells with NS siRNA or IRE1a-targeted siRNAs following 48 h treatment with Gilteritinib (2.5 nM) and ATO (0.5 µM) was tested by western blot. Data are shown as mean ± SD. **P < 0.01

**Fig. 9:**
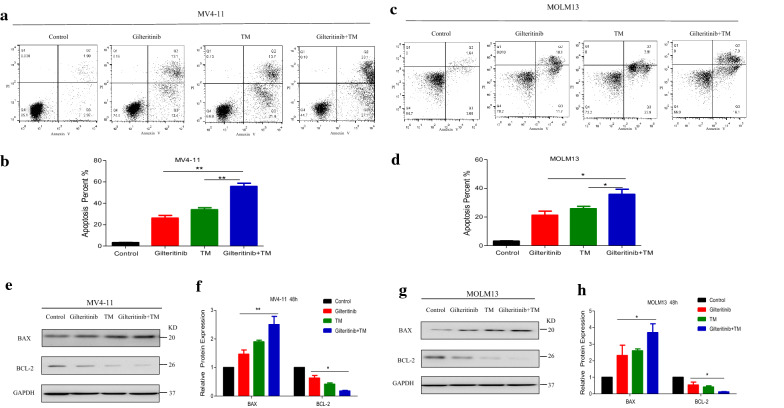
Targeting endoplasmic reticulum stress to cooperate with Gilteritinib promotes apoptosis of FLT3-ITD-mutated cells. **a**–**d** MV4-11 and MOLM13 cells were treated with Gilteritinib (2.5 nM) and/or tunicamycin (TM, 0.2 µM) for 24 h and cell apoptosis was detected using flow cytometry. Data are shown as mean ± SD. *P < 0.05, **P < 0.01. **e**–**h** Expression of BAX and BCL-2 in MV4-11 and MOLM13 cells was detected by western blot following 24 h treatment with Gilteritinib (2.5 nM) and/or TM (0.2 µM). Data are shown as mean ± SD. *P < 0.05, **P < 0.01

To further explore the role of IRE1a on the synergistic effects observed with combined Gilteritinib with ATO treatment, we transiently transfected MV4-11 and MOLM13 with siRNA against IRE1a or nonsilencing (NS) siRNA as a control. Following induction of siRNA expression, the decreased IRE1a mRNA level was corroborated using RT-qPCR (Fig. 8a) and there was a significant reduction in basal IRE1a protein level compared with NS siRNA induction in both FLT3-ITD-mutated cells (Fig. 8b, c). Consequently, when IRE1a siRNA-expressing cells were treated with Gilteritinib (2.5 nM) and ATO (0.5 µM) in combination for 48 h, there was a decrease in Annexin V^+^ cells compared with NS siRNA-expressing cells treated with the same dose of two drugs (Fig. 8d–g). Furthermore, upregulation-trend of pro-apoptotic molecule BAX and downregulation-trend of anti-apoptotic molecule BCL-2 were reversed by knockdown of IRE1a in MV4-11 (Fig. 8h, i) and MOLM13 cells (Fig. 8j, k). Thus, the combined effect of Gilteritinib and ATO was achieved through IRE1a-JNK signal pathway. Furthermore, MV4-11 and MOLM13 cells treated with tunicamycin (TM, 0.2 µM) (an endoplasmic reticulum stress activator) plus Gilteritinib for 24 h had similar effects with those treated with ATO plus Gilteritinib. The increased proportion of apoptotic cells was observed (Fig. 9a–d) and western blot showed an increased BAX expression and a decreased BCL-2 expression after treatment with Gilteritinib plus TM (Fig. 9e–h). Thus, targeting IRE1a in endoplasmic reticulum stress pathway to cooperate with Gilteritinib may serve as a new treatment strategy on FLT3-ITD mutant AML.

### Gilteritinib plus ATO is efficacious in mouse xenograft models of FLT3-ITD mutant AML

**Fig. 10:**
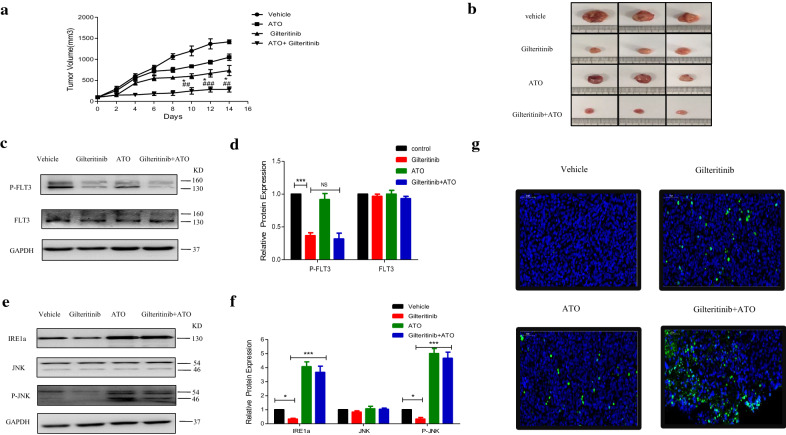
Gilteritinib plus ATO is efficacious in mouse xenograft models of FLT3-ITD mutant AML. **a**, **b** Six-week-old female nude mice were subcutaneously injected in the flank with 1 × 10^7^ MV4-11 cells to establish a xenograft model of AML. When tumors were established, mice were treated with daily vehicle, intraperitoneal injection of ATO (1 mg/kg/day), oral administration of Gilteritinib (10 mg/kg/day), or Gilteritinib (10 mg/kg/day) plus ATO (1 mg/kg/d) in combination for 2 weeks. Tumor growth was evaluated by measuring tumors with a caliper. *P < 0.05, Gilteritinib + ATO group vs the Gilteritinib group. ^##^P < 0.01, ^###^P < 0.001, Gilteritinib + ATO group vs the ATO group. **c**, **d** Tumor cells from vehicle group, Gilteritinib group, ATO group and Gilteritinib + ATO group were lysed and analyzed by western blot using indicated antibodies (P-FLT3 and FLT3). ***P < 0.001. **e**, **f** Tumor cells from vehicle group, Gilteritinib group, ATO group and Gilteritinib + ATO group were lysed and protein levels of IRE1a, JNK and P-JNK were determined by western blot. *P < 0.05, ***P < 0.001. **g** Apoptosis was measured in tumor tissue from vehicle group, Gilteritinib group, ATO group and Gilteritinib + ATO group using the TUNEL assay. Green-fluorescence represents apoptosis

We next explored the efficacy of Gilteritinib plus ATO treatment in mouse xenograft models of AML. We established a subcutaneous xenograft model of AML using 6-week-old female nude mice, which were subcutaneously injected in the flank with MV4-11 cells. When tumors were established, mice were treated with daily intraperitoneal injection of ATO (1 mg/kg/day), oral administration of Gilteritinib (10 mg/kg/day), or Gilteritinib (10 mg/kg/day) plus ATO (1 mg/kg/day) in combination for 2 weeks. After treatment for 2 weeks, the tumors were removed from the nude mice for further experiments. Gilteritinib and ATO in combination strongly inhibited the growth of tumors (Fig. 10a, b). Dose of ATO at 1 mg/kg/day in vivo could not degrade the total protein level of FLT3. Gilteritinib treatment significantly inhibited FLT3 activation (phosphorylated-FLT3) and combinatorial effect on inhibition of FLT3 activation was limited (Fig. 10c, d). Consistent with vitro studies, Gilteritinib plus ATO treatment to activate IRE1a-JNK signal pathway was further confirmed in vivo. Gilteritinib resulted in reduction on expression of both IRE1a and phosphorylated JNK, but cotreatment with ATO led to a significant increase (Fig. 10e, f). The combination therapy had a synergistic antileukemic effect to increase apoptosis as shown in TUNEL staining (Fig. 10g). Taken together, these results suggested that the combination of Gilteritinib plus ATO was efficacious to reduce the tumor volume in mouse xenograft models of FLT3-ITD mutant AML.

## Discussion

Our results first demonstrated that the limited ability of Gilteritinib to activate endoplasmic reticulum stress could be salvaged by ATO at low concentration through activating IRE1a-JNK signal pathway in FLT3-ITD mutant cells.

FLT3-ITD mutations concentrated in the juxtamembrane domain induce ligand-independent dimerization to activate phosphatidylinositol 3-kinase (PI3K)/AKT, mitogen-activated extracellular signal regulated kinase (MEK)/ERK and Janus Kinase (JAK)/STAT signal transduction pathways and achieve cytokine-independent cell proliferation and finally result in leukemia under the synergistic effect of other oncogenes [32]. Compared to the first generation inhibitor sorafenib which inhibits broad-spectrum of fibrosarcoma protein (RAF-1), vascular endothelial growth factor (VEGF), c-KIT, platelet-derived growth factor receptor (PDGFR) and FLT3, Gilteritinib has a strong inhibitory effect on FLT3 and the smallest effect on c-KIT with a higher specificity [37]. Gilteritinib is the second generation of FLT3 TKIs like Crenolanib and Quizartinib. Gilteritinib is more effective in suppression on FLT3-ITD/D835Y mutation than Quizartinib [38] and the therapeutic effect of Crenolanib at the maximum tolerable dose is limited compared to Gilteritinib in mouse model [39]. Thus, Gilteritinib may be more potent than other TKIs in the treatment of FLT3 mutant leukemia. FLT3-ITD positive cells were sensitive to Gilteritinib (Fig. 1c–f). But curative ability of Gilteritinib as a single reagent was limited, combining Gilteritinib with other regimens was needed. Recently, combination of ATO with sorafenib is proved to be effective on inhibition of FLT3-ITD positive cells with a specific exploration for the effect of ATO at high concentration on the degradation of mutant FLT3 protein [27, 28]. But high concentration of ATO increases the occurrence of side effects, even fatal for patients. In our study, combination of Gilteritinib with ATO at low concentration of 0.5 µM could inhibit cell proliferation (Fig. 2a, b), increase apoptosis (Fig. 3a–d), weaken ability of invasion (Fig. 3g, h) and induce cell cycle arrest (Fig. 3i, j). Most previous studies performed investigation on ATO at a high concentration (1 µM, especially 2 µM and 4 µM) that could decrease deubiquitinating enzyme USP10 to contribute to degradation of total FLT3 protein [28], but we observed no obvious reduction of USP10 and total FLT3 protein following treatment with ATO at 0.5 µM (Fig. 4a–d). Gilteritinib dramatically inhibited phosphorylated FLT3 (Fig. 4e–h) and its downstream phosphorylation targets including STAT5 (Figs. 5a, b, 6a, b), AKT (Figs. 5c, d, 6c, d) and ERK (Figs. 5e, f, 6e, f) at concentration of 2.5 nM, further suppression on phosphorylation of FLT3 (Fig. 4e–h) and its downstream phosphorylation targets (Fig. 5a–f, 6a–f) wasn’t observed with addition of ATO at low concentration of 0.5 µM. But the addition of ATO at 0.5 µM to Gilteritinib (2.5 nM) could increase apoptosis, other mechanisms were needed to clarify in the combination of the two drugs.

Previous literatures have revealed that FLT3 TKIs could transfer FLT3 protein from endoplasmic reticulum to cell membrane [40], we confirmed that Gilteritinib as a FLT3 TKI increased 160KD FLT3 protein (Fig. 7a–d). The increase of 160KD FLT3 protein induced by Gilteritinib might lead to inactivation of endoplasmic reticulum stress to weaken the ability of inhibition on FLT3-ITD positive cells. Recent studies indicate that endoplasmic reticulum stress-activated PERK, ATF6 and IRE1a play an important role in apoptosis [41] and sustained IRE1a activation serves as a positive regulatory factor to phosphorylate its downstream target JNK [42]. Detecting the marker proteins of endoplasmic reticulum stress, we found that IRE1a and its downstream phosphorylated JNK were inhibited after Gilteritinib treatment (Fig. 7e–h). This downregulation of IRE1a and phosphorylated JNK was reversed by cotreatment with ATO (Fig. 7i, j). Our experiments showed that ATO at 0.5 µM could potentiate Gilteritinib-induced apoptosis in FLT3-ITD positive leukemic cells with upregulation of both IRE1a and its downstream phosphorylation target JNK. The apoptotic effect of combination of Gilteritinib with ATO on MV4-11 and MOLM13 cells was weakened when IRE1a was knocked down (Fig. 8d–k) to further confirm that the synergistic effect of Gilteritinib plus ATO was achieved by activating IRE1a-JNK signal pathway. In addition, the combination of Gilteritinib with endoplasmic reticulum stress activator TM to increase percentage of apoptosis (Fig. 9a–d) and to activate apoptosis signal pathway (Fig. 9e–h) proved that it was an important target to strengthen endoplasmic reticulum stress in Gilteritinib-treated cells. In mouse xenograft models of FLT3-ITD mutant AML, Gilteritinib alone or ATO alone both reduced tumor size, while combination of Gilteritinib with ATO produced a profound treatment (Fig. 10a, b). IRE1a and phosphorylated JNK were suppressed by Gilteritinib in vivo and cotreatment with ATO produced an increased expression (Fig. 10e, f) to induce further apoptosis (Fig. 10g) under the condition that ATO at 1 mg/kg/day could not degrade FLT3 protein (Fig. 10c, d). The role of phosphorylated JNK in the synergistic effect of combination treatment was consistent with IRE1a in vitro and vivo, and JNK was also an important target of endoplasmic reticulum stress pathway in Gilteritinib-treated FLT3-ITD mutant cells. Though no change of total expression of JNK protein was produced under combination therapy, more exploration on its effect could provide a valuable direction in future research. Gilteritinib alone was more potent to reduce the tumor volume as compared to ATO alone, on the one hand, this may due to the facts that Gilteritinib was far more effective than ATO to target FLT3 signaling; on the other hand, the effect of ATO on reduction of tumor growth may hindered because of low dose applied.

## Conclusion

In conclusion, our study first uncovered the mechanism of combination of the second generation FLT3 TKI Gilteritinib with ATO at low concentration. ATO at low concentration could rescue the defective ability of Gilteritinib on activation of IRE1a-JNK signal pathway in FLT3-ITD mutant cells. Low doses of each drug in combination maximize synergy and possibly reduce toxicity with little side effect in clinic. Our findings may provide a new target for combination treatment and a novel theoretical basis for the treatment of FLT3-ITD positive AML patients. Further clinical trials are needed to confirm the combinatorial effects in the therapy of FLT3-ITD positive AML.

## Supplementary information

**Additional file 1: Figure S1** Analysis on FLT3 (160KD and 130KD) in ATO and Gilteritinib treated MV4-11 and MOLM13 cells. A-B, MV4-11 and MOLM13 cells were treated with ATO (0.5 µM) for 48 h and data of protein levels of FLT3 are shown as the mean ± SD. C, D, MV4-11 and MOLM13 cells were treated with Gilteritinib (2.5 nM) for 48 h and data of protein levels of FLT3 are shown as the mean ± SD. *P < 0.05. **Figure S2.** USP10 protein was not affected by Gilteritinib. A–D, MV4-11 and MOLM13 cells were treated with Gilteritinib (2.5 nM) for 48 h and protein levels of USP10 were determined by western blot. Data are shown as the mean ± SD.

## Data Availability

All data generated or analysed during the present study are included in this published article. The data that support the findings of this study are available on request from the corresponding author.

## Abbreviations

AML: Acute myeloid leukemia
AKT: Protein kinase B
APL: Acute promyelocytic leukemia
ATF6: Activating transcription factor 6
ATO: Arsenic trioxide
BAX: bcl2-associated X protein
BCA: Bicinchoninic acid
Bcl-2: B-cell lymphoma-2
BSA: Bovine serum albumin
DMSO: Dimethyl sulfoxide
ERK: Extracellular regulated protein kinases
FBS: Fetal bovine serum
FDA: Food and Drug Administration
FITC: Fluorescein Isothiocyanate
FLT3: FMS-like tyrosine kinase 3
GAPDH: Glyceraldehyde-3-phosphate dehydrogenase
GRP94: Glucose regulated protein 94
IRE1a: Inositol-requiring enzyme-1a
ITDs: Internal tandem duplications
JAK: Janus Kinase
JNK: c-Jun NH2-terminal kinase
MEK: Mitogen-activated extracellular signal regulated kinase
PBS: Phosphate buffer saline
PDGFR: Platelet-derived growth factor receptor
PERK: PKR-like ER kinase
PML-RARA: Promyelocytic leukemia-retinoic acid receptor-alpha
PI: Propidium iodide
PI3K: Phosphatidylinositol 3-kinase
PVDF: Polyvinylidene fluoride
RAF: Fibrosarcoma protein
RIPA: Radio-Immunoprecipitation Assay
STAT5: Signal transducers and activators of transcription 5
TBST: Tris Buffered Saline Tween
TKD: Tyrosine-kinase domain
TKIs: Tyrosine kinase inhibitors
TM: Tunicamycin
USP10: Human Recombinant Protein
VEGF: Vascular endothelial growth factor

## References

[1] Ferrara F, Schiffer CA (2013). Acute myeloid leukaemia in adults. Lancet.

[2] Falini B, Brunetti L, Martelli MP (2015). Dactinomycin in NPM1-mutated acute myeloid leukemia. N Engl J Med.

[3] Marchwicka A, Cebrat M, Sampath P, Sniezewski L, Marcinkowska E (2014). Perspectives of differentiation therapies of acute myeloid leukemia: the search for the molecular basis of patients’ variable responses to 1,25-dihydroxyvitamin d and vitamin d analogs. Front Oncol.

[4] Maroc N, Rottapel R, Rosnet O, Marchetto S, Lavezzi C, Mannoni P, Birnbaum D, Dubreuil P (1993). Biochemical characterization and analysis of the transforming potential of the FLT3/FLK2 receptor tyrosine kinase. Oncogene.

[5] Koch S, Jacobi A, Ryser M, Ehninger G, Thiede C (2008). Abnormal localization and accumulation of FLT3-ITD, a mutant receptor tyrosine kinase involved in leukemogenesis. Cells Tissues Organs.

[6] Nakao M, Yokota S, Iwai T, Kaneko H, Horiike S, Kashima K, Sonoda Y, Fujimoto T, Misawa S (1996). Internal tandem duplication of the flt3 gene found in acute myeloid leukemia. Leukemia.

[7] Yokota S, Kiyoi H, Nakao M, Iwai T, Misawa S, Okuda T, Sonoda Y, Abe T, Kahsima K, Matsuo Y (1997). Internal tandem duplication of the FLT3 gene is preferentially seen in acute myeloid leukemia and myelodysplastic syndrome among various hematological malignancies. A study on a large series of patients and cell lines. Leukemia.

[8] Frohling S, Schlenk RF, Breitruck J, Benner A, Kreitmeier S, Tobis K, Dohner H, Dohner K (2002). Prognostic significance of activating FLT3 mutations in younger adults (16 to 60 years) with acute myeloid leukemia and normal cytogenetics: a study of the AML Study Group Ulm. Blood.

[9] Abu-Duhier FM, Goodeve AC, Wilson GA, Care RS, Peake IR, Reilly JT (2001). Identification of novel FLT-3 Asp835 mutations in adult acute myeloid leukaemia. Br J Haematol.

[10] Yamamoto Y, Kiyoi H, Nakano Y, Suzuki R, Kodera Y, Miyawaki S, Asou N, Kuriyama K, Yagasaki F, Shimazaki C (2001). Activating mutation of D835 within the activation loop of FLT3 in human hematologic malignancies. Blood.

[11] Konig H, Levis M (2015). Targeting FLT3 to treat leukemia. Expert Opin Ther Targets.

[12] Gorcea CM, Burthem J, Tholouli E (2018). ASP2215 in the treatment of relapsed/refractory acute myeloid leukemia with FLT3 mutation: background and design of the ADMIRAL trial. Future Oncol.

[13] Lee LY, Hernandez D, Rajkhowa T, Smith SC, Raman JR, Nguyen B, Small D, Levis M (2017). Preclinical studies of gilteritinib, a next-generation FLT3 inhibitor. Blood.

[14] Wu M, Li C, Zhu X (2018). FLT3 inhibitors in acute myeloid leukemia. J Hematol Oncol.

[15] Fathi AT, Chen YB (2017). The role of FLT3 inhibitors in the treatment of FLT3-mutated acute myeloid leukemia. Eur J Haematol.

[16] Usuki K, Sakura T, Kobayashi Y, Miyamoto T, Iida H, Morita S, Bahceci E, Kaneko M, Kusano M, Yamada S (2018). Clinical profile of gilteritinib in Japanese patients with relapsed/refractory acute myeloid leukemia: an open-label phase 1 study. Cancer Sci.

[17] Dhillon S (2019). Gilteritinib: first global approval. Drugs.

[18] Perl AE, Altman JK, Cortes J, Smith C, Litzow M, Baer MR, Claxton D, Erba HP, Gill S, Goldberg S (2017). Selective inhibition of FLT3 by gilteritinib in relapsed or refractory acute myeloid leukaemia: a multicentre, first-in-human, open-label, phase 1–2 study. Lancet Oncol.

[19] Estan MC, Calvino E, de Blas E, Boyano-Adanez Mdel C, Mena ML, Gomez-Gomez M, Rial E, Aller P (2012). 2-Deoxy-d-glucose cooperates with arsenic trioxide to induce apoptosis in leukemia cells: involvement of IGF-1R-regulated Akt/mTOR, MEK/ERK and LKB-1/AMPK signaling pathways. Biochem Pharmacol.

[20] Breccia M, Lo-Coco F (2011). Gemtuzumab ozogamicin for the treatment of acute promyelocytic leukemia: mechanisms of action and resistance, safety and efficacy. Expert Opin Biol Ther.

[21] Akao Y, Yamada H, Nakagawa Y (2000). Arsenic-induced apoptosis in malignant cells in vitro. Leuk Lymphoma.

[22] Shen ZY, Shen J, Cai WJ, Hong C, Zheng MH (2000). The alteration of mitochondria is an early event of arsenic trioxide induced apoptosis in esophageal carcinoma cells. Int J Mol Med.

[23] Chiu HW, Tseng YC, Hsu YH, Lin YF, Foo NP, Guo HR, Wang YJ (2015). Arsenic trioxide induces programmed cell death through stimulation of ER stress and inhibition of the ubiquitin-proteasome system in human sarcoma cells. Cancer Lett.

[24] Qiu Y, Dai Y, Zhang C, Yang Y, Jin M, Shan W, Shen J, Lu M, Tang Z, Ju L (2018). Arsenic trioxide reverses the chemoresistance in hepatocellular carcinoma: a targeted intervention of 14-3-3eta/NF-kappaB feedback loop. J Exp Clin Cancer Res CR.

[25] Lauwers GY, Scott GV, Karpeh MS (1995). Immunohistochemical evaluation of bcl-2 protein expression in gastric adenocarcinomas. Cancer.

[26] Zhang TC, Cao EH, Li JF, Ma W, Qin JF (1999). Induction of apoptosis and inhibition of human gastric cancer MGC-803 cell growth by arsenic trioxide. Eur J Cancer.

[27] Wang R, Li Y, Gong P, Gabrilove J, Waxman S, Jing Y (2018). Arsenic trioxide and sorafenib induce synthetic lethality of FLT3-ITD acute myeloid leukemia cells. Mol Cancer Ther.

[28] Nagai K, Hou L, Li L, Nguyen B, Seale T, Shirley C, Ma H, Levis M, Ghiaur G, Duffield A (2018). Combination of ATO with FLT3 TKIs eliminates FLT3/ITD + leukemia cells through reduced expression of FLT3. Oncotarget.

[29] Masciarelli S, Capuano E, Ottone T, Divona M, Lavorgna S, Liccardo F, Sniegocka M, Travaglini S, Noguera NI, Picardi A (2019). Retinoic acid synergizes with the unfolded protein response and oxidative stress to induce cell death in FLT3-ITD + AML. Blood Adv.

[30] Chen Y, Wang J, Hong DY, Chen L, Zhang YY, Xu YN, Pan D, Fu LY, Tao L, Luo H (2017). Baicalein has protective effects on the 17beta-estradiol-induced transformation of breast epithelial cells. Oncotarget.

[31] Jia X, Zhang Z, Luo K, Zheng G, Lu M, Song Y, Liu H, Qiu H, He Z (2017). TCRP1 transcriptionally regulated by c-Myc confers cancer chemoresistance in tongue and lung cancer. Sci Rep.

[32] Takahashi S (2011). Downstream molecular pathways of FLT3 in the pathogenesis of acute myeloid leukemia: biology and therapeutic implications. J Hematol Oncol.

[33] Schmidt-Arras DE, Bohmer A, Markova B, Choudhary C, Serve H, Bohmer FD (2005). Tyrosine phosphorylation regulates maturation of receptor tyrosine kinases. Mol Cell Biol.

[34] Li X, Zhu H, Huang H, Jiang R, Zhao W, Liu Y, Zhou J, Guo F-J (2012). Study on the effect of IRE1α on cell growth and apoptosis via modulation PLK1 in ER stress response. Mol Cell Biochem.

[35] So JS (2018). Roles of endoplasmic reticulum stress in immune responses. Mol Cells.

[36] Wu XY, Fan RT, Yan XH, Cui J, Xu JL, Gu H, Gao YJ (2015). Endoplasmic reticulum stress protects human thyroid carcinoma cell lines against ionizing radiation-induced apoptosis. Mol Med Rep.

[37] Grunwald MR, Levis MJ (2015). FLT3 tyrosine kinase inhibition as a paradigm for targeted drug development in acute myeloid leukemia. Semin Hematol.

[38] Smith CC, Wang Q, Chin CS, Salerno S, Damon LE, Levis MJ, Perl AE, Travers KJ, Wang S, Hunt JP (2012). Validation of ITD mutations in FLT3 as a therapeutic target in human acute myeloid leukaemia. Nature.

[39] Zimmerman EI, Turner DC, Buaboonnam J, Hu S, Orwick S, Roberts MS, Janke LJ, Ramachandran A, Stewart CF, Inaba H (2013). Crenolanib is active against models of drug-resistant FLT3-ITD-positive acute myeloid leukemia. Blood.

[40] Reiter K, Polzer H, Krupka C, Maiser A, Vick B, Rothenberg-Thurley M, Metzeler KH, Dorfel D, Salih HR, Jung G (2018). Tyrosine kinase inhibition increases the cell surface localization of FLT3-ITD and enhances FLT3-directed immunotherapy of acute myeloid leukemia. Leukemia.

[41] Ma B, Zhang H, Wang Y, Zhao A, Zhu Z, Bao X, Sun Y, Li L, Zhang Q (2018). Corosolic acid, a natural triterpenoid, induces ER stress-dependent apoptosis in human castration resistant prostate cancer cells via activation of IRE-1/JNK, PERK/CHOP and TRIB3. J Exp Clin Cancer Res CR.

[42] Yamamoto K, Ichijo H, Korsmeyer SJ (1999). BCL-2 is phosphorylated and inactivated by an ASK1/Jun N-terminal protein kinase pathway normally activated at G(2)/M. Mol Cell Biol.

