# Supplementary material for: Arsenic trioxide potentiates Gilteritinib-induced apoptosis in FLT3-ITD positive leukemic cells via IRE1a-JNK-mediated endoplasmic reticulum stress
Source: Cancer Cell Int. 2020 Jun 17;20:250. doi: 10.1186/s12935-020-01341-5 (PMC7298957; doi:10.1186/s12935-020-01341-5)
Supplement: Supplementary file 1 — Additional file 1: Figure S1 Analysis on FLT3 (160KD and 130KD) in ATO and Gilteritinib treated MV4-11 and MOLM13 cells. A-B, MV4-11 and MOLM13 cells were treated with ATO (0.5 µM) for 48 h and data of protein levels of FLT3 are shown as the mean ± SD. C, D, MV4-11 and MOLM13 cells were treated with Gilteritinib (2.5 nM) for 48 h and data of protein levels of FLT3 are shown as the mean ± SD. *P < 0.05. Figure S2. USP10 protein was not affected by Gilteritinib. A–D, MV4-11 and MOLM13 cells were treated with Gilteritinib (2.5 nM) for 48 h and protein levels of USP10 were determined by western blot. Data are shown as the mean ± SD. [file 12935_2020_1341_MOESM1_ESM.pdf]

Figure S1

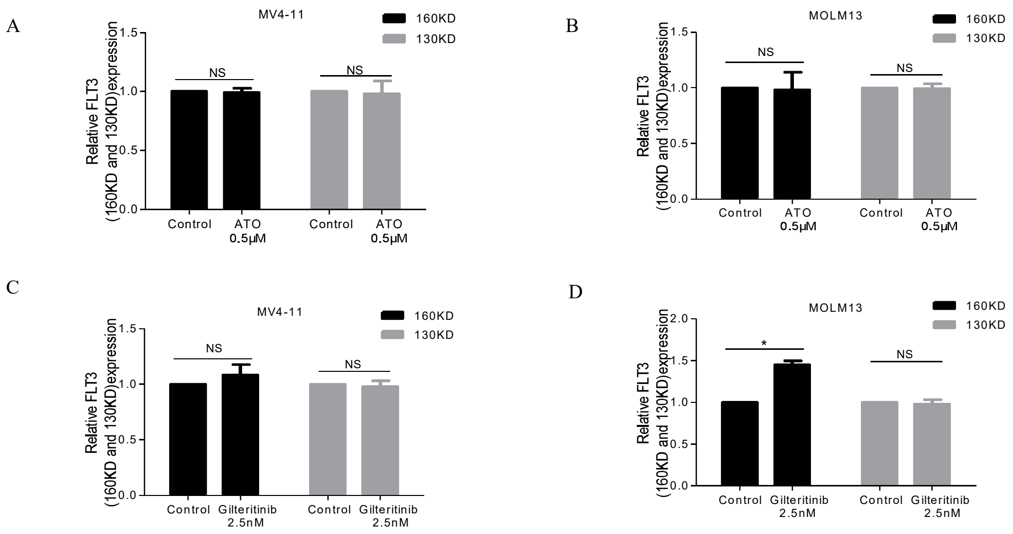
A
MV4-11
160KD
130KD
Relative FLT3 (160KD and 130KD)expression
1.5
1.0
0.5
0.0
NS
NS
Control
ATO 0.5μM
Control
ATO 0.5μM
B
MOLM13
160KD
130KD
Relative FLT3 (160KD and 130KD)expression
1.5
1.0
0.5
0.0
NS
NS
Control
ATO 0.5μM
Control
ATO 0.5μM
C
MV4-11
160KD
130KD
Relative FLT3 (160KD and 130KD)expression
1.5
1.0
0.5
0.0
NS
NS
Control
Gilteritinib 2.5nM
Control
Gilteritinib 2.5nM
D
MOLM13
160KD
130KD
Relative FLT3 (160KD and 130KD)expression
2.0
1.5
1.0
0.5
0.0
*
NS
Control
Gilteritinib 2.5nM
Control
Gilteritinib 2.5nM

Fig. S1 Analysis on FLT3 of 160KD and 130KD in ATO and Gilteritinib treated MV4-11 and MOLM13 cells. A-B MV4-11 and MOLM13 cells were treated with ATO (0.5μM) for 48h and data of protein levels of FLT3 are shown as the mean±SD. **C-D** MV4-11 and MOLM13 cells were treated with Gilteritinib (2.5nM) for 48h and data of protein levels of FLT3 are shown as the mean±SD. *P<0.05.

Figure S2

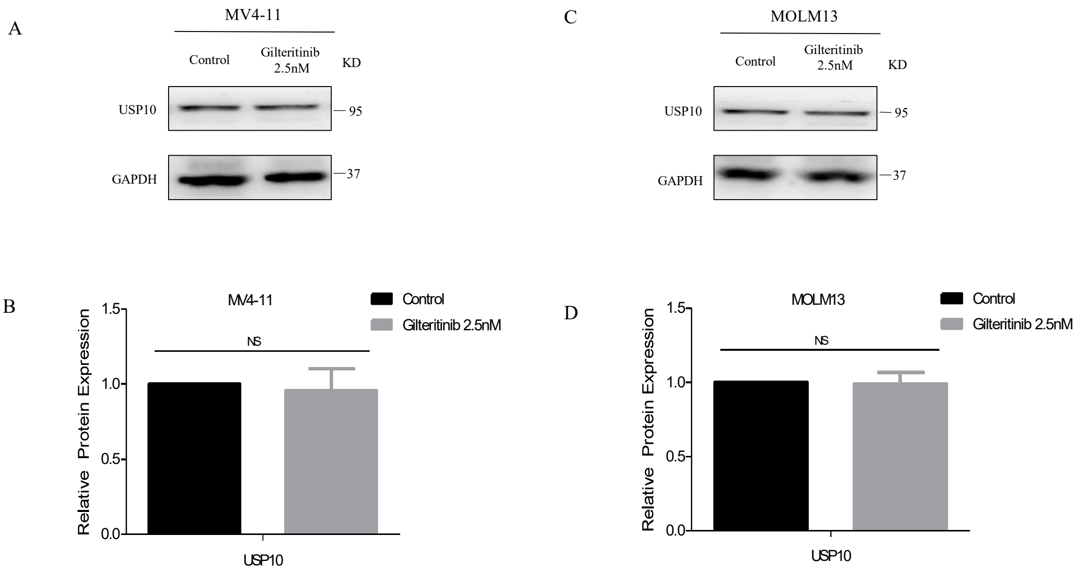

A
MV4-11
Control
Gilteritinib 2.5nM
KD
USP10
95
GAPDH
37
C
MOLM13
Control
Gilteritinib 2.5nM
KD
USP10
95
GAPDH
37
B
MV4-11
Control
Gilteritinib 2.5nM
Relative Protein Expression
1.5
1.0
0.5
0.0
NS
USP10
D
MOLM13
Control
Gilteritinib 2.5nM
Relative Protein Expression
1.5
1.0
0.5
0.0
NS
USP10

Fig. S2 USP10 protein was not affected by Gilteritinib. A-D, MV4-11 and MOLM13 cells were treated with Gilteritinib (2.5nM) for 48h and protein levels of USP10 were determined by western blot. Data are shown as the mean±SD.
